# Reliability and validity of a semi-quantitative food frequency questionnaire: dietary intake assessment among multi-ethnic populations in Northwest China

**DOI:** 10.1186/s41043-023-00452-9

**Published:** 2023-10-19

**Authors:** Leilei Zhai, Huiyue Pan, Hanqi Cao, Shupeng Zhao, Ping Yao

**Affiliations:** The First Department of Gastroenterology, The First Affiliated Hospital of Xinjiang Medical University, No.393, Xinyi Road, Urumqi, 830011 Xinjiang Uygur Autonomous Region China

**Keywords:** Reproducibility, Validity, Food frequency questionnaire, Dietary assessment, Multi-ethnic populations

## Abstract

**Background:**

Few multi-ethnic dietary culture-sensitive food frequency questionnaires (FFQ) have been developed due to the complexity and diversity of cooking methods and styles. This study aimed to develop and validate a specific FFQ among multi-ethnic groups in Northwest China.

**Methods:**

In the reliability study, 139 participants aged 20–65 completed two identical FFQs separated by 3 months. The relative validation of the FFQ was assessed by three 24-h recalls (24HR) employed in the interval of two FFQs, as a reference. Stratified analyses were also conducted by the major ethnic groups (*Han* nationality or Ethnic minority).

**Results:**

For reproducibility, the median (range) of Spearman’s correlation coefficients (SCC) was 0.71 (0.43–0.84) for nutrients. The intra-class correlation coefficients (ICC) covered a spectrum from 0.39 to 0.78 (median: 0.64). Meanwhile, the weighted kappa values ranged from 0.11 to 0.64. For validity, the median (range) of Pearson’s correlation coefficients derived from the energy unadjusted and the adjusted values between FFQ and 24HR were 0.61 (0.12–0.79) and 0.56 (0.12–0.77), respectively. The results of correlation coefficients were similar between the two ethnic groups. Moreover, the Bland–Altman plots likewise demonstrated a satisfactory level of agreement between the two methods.

**Conclusions:**

The FFQ showed acceptable reproducibility and moderate relative validity for evaluating dietary intake among multi-ethnic groups in northwest China. It could be a credible nutritional screening tool for forthcoming epidemiological surveys of these populations.

**Supplementary Information:**

The online version contains supplementary material available at 10.1186/s41043-023-00452-9.

## Introduction

Noncommunicable and chronic diseases (NCDs) are increasing in prevalence and now giving rise to a tremendous burden on the global healthcare system [1]. Dietary and nutritional approaches are of paramount importance in the management of NCDs, impacting health status throughout the life course [2]. Hence, comprehensive studies on dietary-related exploration are essential in improving health and well-being of all nations and races [3]. Compared to other dietary evaluation methods, the food frequency questionnaire (FFQ) is the most cost-effective and practical tool for nutritional epidemiology studies [4]. Multiple FFQs had been designed for distinct populations that span the region, gender, age, and even the degree of socialization in China [5–13]. To our knowledge, the data of multi-ethnic dietary cultures-sensitive FFQs developed for dietary intake assessment among specific populations were limited. There is a certain heterogeneity in dietary habits among these distinct ethnic groups due to diverse cultural backgrounds.

Xinjiang province locates in northwest China, as a representative area of multi-ethnic populations gathered, whose population structure is mainly composed of *Han*, *Uygur*, and *Kazak* nationality (account for more than 90% of the total population). According to surveillance data across the Xinjiang multi-ethnic cohort study (XMC), the prevalence of NCDs substantially varies by ethnic group but is higher than the national average in 2018 [14]. Ethnically diversity accompanies variety in diet habits and patterns, which may give the continuing rise in chronic disease prevalence. Previous FFQs [11, 15] based on sole ethnicity cannot guarantee accurate responses in multi-ethnic settings since FFQs should be tailored to the target populations [16]. Further studies should focus on developing and evaluating a culture-sensitive FFQ to optimize dietary intake assessment for proposing effective targeted interventions. Low recall bias is indispensable for a feasible questionnaire [17]. The reproducibility and validity of the FFQ must be examined for the specific populations in dietary-related studies [18]. In this study, we designed a novel and comprehensive semi-quantitative food frequency questionnaire (FFQ) that accounts for traditional ethnic-specific foods and local mixed cuisines and assessed its reliability and validity among the Xinjiang multi-ethnic natural populations.

## Materials and methods

### Participants

**Fig. 1:**
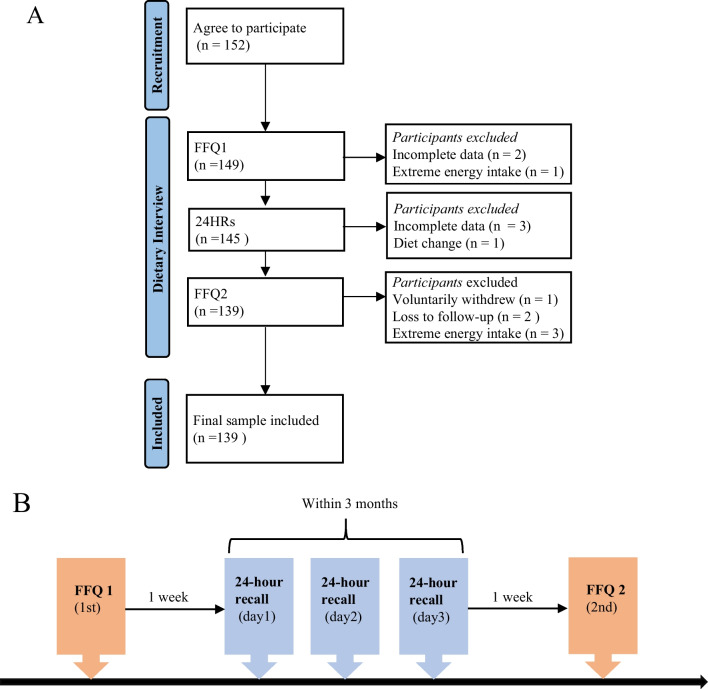
Flowchart of the study. **A** Sample selection flow diagram. **B** Sequence of dietary assessment measurements (FFQ: food frequency questionnaire, 24HR: 24-h recall)

The diet assessment was conducted among residents who had lived in the region of Xinjiang province for more than 5 years. The included researchers were people who underwent physical examination in the First Affiliated Hospital of Xinjiang Medical University. We will not apply restrictions on ethnic backgrounds. Those who were able to receive telephone interviews and complete questionnaires were included, with the exception of pregnant and lactating women, or people with malignant and chronic diseases (e.g., type 2 diabetes or celiac disease) who required strict dietary control, and all participants were older than 18 years and no more than 65 years old. A total of 152 participants were recruited from September 2021 to October 2022. Thirteen individuals were excluded for personal reasons including incomplete data (*n* = 5), diet change (*n* = 1), voluntarily withdrew (*n* = 1), and participants who had extreme total energy intake values ≤ 600 or ≥ 4500 kcal [19] (FFQ1, *n* = 1; FFQ2, *n* = 3), and loss to follow-up (*n* = 2) in the diet administrations (shown in Fig. 1A). In total, 139 who completed this study were taken into the final analysis.

### Dietary intake assessment

Dietary intake was measured via the interviewer-administered FFQ and three 24-h recalls (24HR). The first interview lasted around 30–40 min conducted at the medical examination site and was divided into two parts. In the first step, demographic and anthropometric data were collected for all participants. Demographic and lifestyle variables of age, gender, ethnicity, smoking status, alcohol consumption, education levels, and monthly income were obtained by a structured questionnaire. Besides, anthropometric measurements were obtained from physical examinations. The height and weight were obtained from physical examinations, and body mass index (BMI) was calculated as a ratio of weight (kg) to the square of height (m^2^). Secondly, the first FFQ (FFQ1) was performed by two trained interviewers face-to-face. The same participants were invited to participate in the second interview (FFQ2) approximately three months later at the selected location of their convenience. During the study period, all three datasets of the 24HRs were conducted over the course of three non-consecutive days (two weekdays and one weekend day) through a phone interview. The sequence of dietary assessment measurements is shown in Fig. 1B. This study was approved by the Institutional Ethics Committee of Xinjiang Medical University (Approval Number: K202202-13) and the Declaration of Helsinki ethical standards had been followed. All participants involved in this study obtained formal informed consent before being interviewed.

### FFQ

The FFQ in this study was modified based on an FFQ designed and validated among Uygur subjects in Urumqi and Ili areas. Combining the Chinese dietary guidelines [20] with local ethnic-specific foods, several items were revised or increased due to changes in dietary characteristics including bacon, pickled vegetables, butter, Cantonese sausage, and barbeque sauce. The final list of food items (*n* = 109) included in this FFQ (shown in Additional file 1: Table S1) was categorized into12 food groups: cereals, coarse grains, vegetables, fungi, fruits, meat and aquatic products, eggs and dairy products, beans, nuts, drinks, snacks, and condiments. Five possible frequency options range from “daily,” “weekly,” “monthly,” and “per year” to “never.” Each item was measured with standard portion size, and food models represented standard portions for the majority of the food items were created to assist individuals in estimating their daily intake. It included almost all typical main foods consumed by the local residents, which were implemented by qualified interviewers. Participants were required to report their consumption frequency and quantity of food items over the past six months. The elders were assisted by accompanying person to make a response.

### 24HR

The relative validity was assessed by comparing dietary intake using FFQ against 24HR, as reference [21]. All participants were requested to recall all food consumed over the course of three non-consecutive days (two weekdays and one weekend day), during the interval of administration of FFQs. The participants received detailed instructions on how to perform dietary recalls at the beginning of enrollment. They were asked to recollect and estimate the portion sizes of all food items (including mixed dishes, recipes, ingredients, beverages, and cooking methods) they had recently consumed during the previous 24 h (midnight-midnight).

### Nutrient calculation

Combining food models and atlases with common tableware helped to estimate portion sizes more accurately (serving bowls, plates, and utensils of standard size) [22, 23]. The “hand” was taken as a reference to facilitate the respondents to judge their food intake appropriately. Additionally, data on dietary preferences, time and location of consumption (at home or outside), recipe components, and cooking methods were gathered. Average daily dietary intake (g/day) was calculated via the amount of each item consumed (g) and multiplied by the number of times “per day.” Based on the number of family members, oil and other recipe ingredients amounts were divided by the proportion of home meals consumed in the FFQ. For nutrients analysis, the precise data of energy and nutrients intake were calculated by a nutritional calculator (version 2.8.0) authorized by the organization of the Chinese Center for Disease Control and Prevention, according to the standard basis of the Chinese Food Composition Database [24]. Any incomplete or incoherent information would be removed. Two trained investigators double entered the primary data acquired into Microsoft Excel for statistical analysis after thoroughly reviewing.

### Statistical analysis

Firstly, the continuous variables with a normal distribution were described by the mean and standard deviation (SD) according to the Kolmogorov–Smirnov tests. Data with non-normal distribution were reported as median and the 25th/75th percentile (P25, P75). Analyses were stratified by ethnicity (*Han* nationality or Ethnic minorities). Chi-squared test was used to compare the categorical variables, and parametric continuous variables of characteristics (age, BMI, weight, and height) were compared across groups with independent t tests.

All dietary data were expressed as medians (P25, P75) because most values were not normal distributions. For reproducibility, differences in nutrients intake between the FFQ1 and FFQ2 were evaluated by Spearman’s correlation coefficient (SCC). The SCC of less than 0.20, 0.20–0.49, and more than 0.50 represent poor, acceptable, and good, respectively [25]. Intra-class correlation coefficients (ICC) analysis and their respective 95% confidence intervals (CI) were calculated to assess the strength of association at the individual level. The degree of agreement for the classification of energy and nutrients intake into quartiles was valued by the weighted kappa statistic.

To assess the validation, Pearson correlation coefficients were assessed for unadjusted and adjusted nutrients between the average intakes of FFQs and 24HRs, respectively. Before regression analysis for energy adjustment, all dietary intake data were log-transformed to accord with normal distribution. Using the residual method of Willett [26] to decrease the influence of the relative correlation between each nutrient and total energy intake, in which residuals were computed from a regression model (regressing nutrient intake on total energy intake). We conducted subgroup analyses separated by ethnicity. Likewise, Bland–Altman analysis [27] was carried out to estimate the agreement of the dietary variables between the two measurements. SPSS (version 25.0, 2017, IBM SPSS Inc) was used for statistical data analysis. A two-sided *P*-value ≤ 0.05 was considered statistically significant.

## Results

### Participants characteristics

There were a total of 152 patients who agreed to take part in this study. During the measurement, 8.6% of participants dropped out. Therefore, the final statistical sample consisted of 139 participants (61 women and 78 men), who completed two FFQs and three 24HRs were included in the following analysis. The baseline characteristics of the participants are shown in Table 1. The sample included participants from a wide range of ages and ethnic backgrounds. The mean (SD) age was 44.94 (11.62) years, deviating from 20 to 65 years old. Ethnicity was evenly distributed in the sample, which was well represented in multi-ethnic groups in Xinjiang. Participants were generally well educated with roughly half (46.0%) having college-level education. A higher proportion of ethnic minority groups (*n* = 77) than Han groups (*n* = 62) were male (58.4% *vs.* 53.2%) and drinkers (46.8% *vs.* 40.3%). Additionally, there was no statistical difference between demographic characteristics (age, BMI, education level, etc.) between *Han* and ethnic minority groups.

**Table 1:** Demographic characteristics of the study populations

Item	Overall	*Han* nationality	Ethnic minority	*P*
	(*n* = 139)	(*n* = 62)	(*n* = 77)	
Age (years)^§^	44.94 (11.62)	43.55 (9.42)	46.05 (10.84)	0.212
Sex (*n*, %)				0.538
Male	78 (56.1)	33 (53.2)	45 (58.4)	
Female	61 (43.9)	29 (46.8)	32 (41.6)	
Ethnicity (*n*, %)				
Han	62 (44.6)			
Uygur	41 (29.5)			
Kazak	31 (22.3)			
Others	5 (3.6)			
Salary (yuan/month) (*n*, %)				0.223
< 2000	46 (38.3)	19 (46.3)	27 (34.2)	
2000–5000	30 (9.2)	17 (12.2)	13 (7.6)	
> 5000	63 (52.5)	27 (41.5)	36 (58.2)	
Smoking (*n*, %)				0.999
No	63 (45.3)	28 (45.2)	35 (45.5)	
Current	58 (41.7)	26 (41.9)	32 (41.6)	
Former	18 (12.9)	8 (12.9)	10 (13.0)	
Alcohol (*n*, %)				0.746
No	65 (46.8)	31 (50.0)	34 (44.2)	
Current	61 (43.9)	25 (40.3)	36 (46.8)	
Former	13 (9.4)	6 (9.7)	7 (9.1)	
Height (m)^§^	1.68 (0.12)	1.69 (0.16)	1.67 (0.09)	0.435
Weight (kg)^§^	66.05 (10.2)	61.58 (8.71)	69.65 (11.32)	0.066
Highest education level (*n*, %)				0.278
Primary school	18 (12.9)	5 (8.1)	13 (16.9)	
Junior high school	21 (15.1)	9 (14.5)	12 (15.6)	
Senior high school	36 (25.9)	20 (32.3)	16 (20.8)	
College	64 (46.0)	28 (45.2)	36 (46.8)	
BMI (kg/m^2^) ^§^	23.33 (2.68)	21.48 (2.15)	24.82 (2.54)	0.096

T test for continuous variables

Chi-squared test for categorical variables

^§^Values are mean (SD), SD: Standard deviation, BMI: Body mass index

### Reproducibility

The median intake of micronutrients and macronutrients derived from FFQ1 was approximately equal to those from FFQ2, which are presented in Table 2. The nutrients were generally slightly lower assessed by FFQ2 than FFQ1, except for energy, protein, vitamin A, calcium, phosphorus, potassium, zinc, selenium, and choline. The SCC for nutrients ranged from 0.43 (magnesium) to 0.84 (energy), (median = 0.71) for nutrients. ICC for nutrients ranged from 0.39 (magnesium 95% CI 0.21, 0.49) to 0.78 (energy 95% CI 0.69, 0.81), while the median of the weighted kappa score (*κw*) was 0.39. All correlations were statistically significant (*P* < 0.05).

**Table 2:** Reproducibility of energy and nutrient intake between FFQ1 and FFQ2 (*n* = 139)

Nutrient	FFQ1		FFQ2		ICC	Kappa	SCC
	Median	P25, P75	Median	P25, P75	95% CI		
Energy (Kcal)	2270.1	2101.3, 2481.9	2283.8	2089.8, 2508.1	0.78 (0.69, 0.81)	0.49**	0.84**
Protein (g)	64.3	59.5, 67.1	65.3	58.1, 71.2	0.69 (0.58, 0.78)	0.33**	0.74**
Fat (g)	72.5	68.1, 75.5	70.8	66.1, 79.2	0.78 (0.70, 0.84)	0.36**	0.78**
Carbohydrates (g)	344.5	328.6, 357.5	337.4	311.6, 369.3	0.59 (0.46, 0.70)	0.25**	0.67**
Dietary fiber (g)	14.0	12.5, 15.0	13.5	10.3, 16.0	0.43 (0.27, 0.57)	0.12*	0.49**
Cholesterol (mg)	270.8	254.1, 292.0	263.0	240.7, 303.8	0.71 (0.63, 0.82)	0.51**	0.77**
Vitamin A (μg RE)	544.5	465.9, 579.4	546.0	441.2, 597.4	0.77 (0.65, 0.80)	0.52**	0.81**
Vitamin D (μg)	10.5	8.5, 13.0	8.5	6.0, 12.3	0.51 (0.25, 0.67)	0.30**	0.63**
Vitamin E (mg)	13.5	11.5, 15.0	12.5	9.6, 13.8	0.45 (0.30, 0.58)	0.18*	0.51**
Vitamin K (μg)	67.8	60.0, 75.5	65.8	50.8, 76.9	0.69 (0.56, 0.79)	0.41**	0.83**
Thiamin (mg)	0.9	0.7, 1.1	0.7	0.5, 1.1	0.74 (0.62, 0.82)	0.24**	0.68**
Riboflavin (mg)	0.9	0.7, 1.2	0.8	0.7, 1.0	0.65 (0.48, 0.77)	0.37**	0.71**
Vitamin B6 (mg)	1.1	0.8, 1.3	1.0	0.7, 1.1	0.60 (0.45, 0.71)	0.36**	0.64**
Vitamin B12 (μg)	1.6	1.1, 2.1	1.3	0.5, 2.2	0.71 (0.62, 0.82)	0.36**	0.81**
Vitamin C (mg)	81.8	73.5, 92.9	57.3	44.6, 82.6	0.43 (-0.07, 0.72)	0.29**	0.70**
Folate (μg)	382.5	331.5, 425.3	379.3	296.9, 466.9	0.72 (0.62, 0.80)	0.43**	0.66**
Calcium (mg)	612.0	513.8, 714.4	619.5	446.3, 724.9	0.76 (0.66, 0.83)	0.64**	0.81**
Phosphorus (mg)	716.0	644.5, 834.5	740.3	592.3, 882.3	0.73 (0.63, 0.80)	0.40**	0.82**
Potassium (mg)	1767.8	1676.5, 1979.4	1612.3	1427.9, 1934.9	0.71 (0.57, 0.80)	0.51**	0.79**
Sodium (mg)	1662.5	1528.1, 1832.8	1503.8	1299.1, 1838.1	0.61 (0.54, 0.74)	0.46**	0.73**
Magnesium (mg)	289.5	276.1, 311.4	256.5	223.9, 289.4	0.39 (0.21, 0.49)	0.21**	0.43**
Iron (mg)	12.5	10.1, 13.5	12.5	9.5, 15.3	0.61 (0.35, 0.74)	0.57**	0.76**
Iodine (μg)	86.0	74.0, 105.9	82.0	57.5, 125.8	0.68 (0.57, 0.77)	0.46**	0.76**
Zinc (mg)	11.5	10.5, 13.5	12.5	9.5, 15.6	0.51 (0.36, 0.63)	0.35**	0.65**
Selenium (μg)	58.5	53.0, 68.0	61.3	54.5, 70.8	0.48 (0.39, 0.61)	0.52**	0.64**
Copper (mg)	1.0	0.9, 1.2	0.7	0.5, 1.2	0.43 (0.24, 0.58)	0.11*	0.55**
Niacinamide (mg)	273.5	260.5, 288.5	256.5	238.1, 279.3	0.63 (0.51, 0.73)	0.22**	0.57**
Choline (mg)	430.5	364.1, 476.5	445.3	314.6, 514.1	0.57 (0.46, 0.68)	0.39**	0.62**

Daily intakes of nutrients estimated by two FFQs; P25, P75, 25th–75th percentile

ICCs, intra-class correlation coefficients; CI, confidence interval; SCC, Spearman correlation coefficients

Statistical significance was accepted with *P < 0.05 (2-tailed). **P < 0.01

### Validity

As shown in Table 3, the median energy intakes (kcal) estimated by the FFQs and 24HRs were 2273.5 kcal and 2195.2 kcal, respectively. The daily intakes of nutrients estimated by the average of 24HRs tended to show slightly less than those from FFQs, for under-estimating on energy, protein, fiber, vitamin D, riboflavin phosphorus, iron, selenium, choline, etc. Energy adjustment was performed using the residual method, residuals were computed from a regression model [26]. Energy adjustment ascended the Pearson correlation coefficients for partial nutrients but descended for majorities of other nutrients. Folate decreased from 0.57 to 0.52, while vitamin B6 was elevated by 0.03. The most obvious changes compared with the unadjusted values occurred in vitamin D (from 0.77 to 0.65). The unadjusted and adjusted validation values for nutrients ranged from 0.12 ~ 0.79 and 0.12 ~ 0.77, respectively. All correlation coefficients between energy unadjusted and adjusted were significant (*P* < 0.05), except for iron and copper (*P* > 0.05).

**Table 3:** Validation of energy and nutrients intakes between FFQs and 24HRs (*n* = 139)

Nutrient	FFQs		24HRs		Pearson correlation coefficients	
	Median	P25, P75	Median	P25, P75	unadjusted ^†^	Energy adjusted^‡^
Energy (Kcal)	2273.5	2100.7, 2505.3	2195.2	2034.3, 2277.1	0.79**	
Protein (g)	64.5	60.0, 69.5	63.1	59.3, 67.2	0.57**	0.56**
Fat (g)	72.2	67.1, 77.3	72.9	68.1, 76.4	0.71**	0.68**
Carbohydrates (g)	340.5	321.3, 365.4	346.5	325.3, 365.2	0.43**	0.38**
Dietary fiber (g)	14.2	11.3, 15.3	14.0	11.2, 16.3	0.26**	0.26**
Cholesterol (mg)	265.5	250.3, 297.5	278.2	257.0, 298.8	0.77**	0.75**
Vitamin A (μg RE)	544.2	456.5, 587.8	551.0	468.3, 577.5	0.61**	0.58**
Vitamin D (μg)	11.5	9.3, 13.6	11.4	9.2, 14.5	0.77**	0.65**
Vitamin E (mg)	13.4	11.5, 15.6	14.2	11.3, 16.7	0.32**	0.28**
Vitamin K (μg)	67.3	55.2, 77.3	68.2	64.9, 77.8	0.60**	0.55**
Thiamin (mg)	0.8	0.6, 1.1	1.1	0.7, 1.3	0.75**	0.76**
Riboflavin (mg)	0.9	0.7, 1.2	0.9	0.7, 1.2	0.75**	0.77**
Vitamin B6 (mg)	1.0	0.7, 1.2	1.2	0.8, 1.4	0.73**	0.76**
Vitamin B12 (μg)	1.3	0.7, 2.1	1.7	1.2, 2.2	0.66**	0.63**
Vitamin C (mg)	67.5	58.3, 87.2	95.4	84.9, 104.2	0.48**	0.49**
Folate (μg)	384.8	331.8, 442.8	386.5	334.8, 457.5	0.57**	0.52**
Calcium (mg)	598.5	491.3, 709.6	629.6	520.3, 705.3	0.64**	0.63**
Phosphorus (mg)	729.5	610.3, 867.8	717.8	639.5, 799.5	0.61**	0.60**
Potassium (mg)	1681.5	1567.3, 1983.8	1876.3	1786.5, 1995.5	0.63**	0.61**
Sodium (mg)	1564.2	1474.8, 1849.8	1763.5	1645.5, 1894.3	0.61*	0.61*
Magnesium (mg)	273.4	255.8, 296.1	309.5	286.3, 336.8	0.21*	0.21*
Iron (mg)	13.2	10.2, 14.7	12.5	9.0, 15.3	0.12	0.12
Iodine (μg)	86.8	66.3, 113.2	87.3	77.4, 97.8	0.49*	0.47*
Zinc (mg)	12.3	10.3, 14.4	11.5	10.3, 14.2	0.22*	0.22*
Selenium (μg)	59.3	54.7, 69.8	58.3	53.5, 68.4	0.62**	0.61**
Copper (mg)	0.8	0.6, 1.2	1.1	0.9, 1.3	0.15	0.14
Niacinamide (mg)	267.4	251.2, 280.5	281.5	263.3, 295.3	0.42**	0.41**
Choline (mg)	445.2	337.5, 493.5	432.6	366.3, 466.2	0.55**	0.53**

Daily intakes of nutrients estimated by FFQs and 24HRs; P25, P75, 25th–75th percentile

^†^Based on log-transformed values

^‡^Energy adjusted using the residual method

Statistical significance was accepted with *P < 0.05, **P < 0.01

### Ethnicity subgroup analysis

Results of reproducibility and validity and mean dietary intake for all nutrients were estimated separately by ethnicity. As shown in Additional file 1: Table S2, among *Han* groups, for reliability, SCC ranged from 0.35 to 0.86 (median = 0.64). For validations, the Pearson correlation coefficients between the two methods were from 0.11 to 0.79 (median = 0.53). Among ethnic minority group, SCC ranged from 0.33 to 0.79 (median = 0.62), the Pearson correlation coefficients between FFQ and 24 HRs ranged from 0.12 to 0.78 (median = 0.57) (shown in Additional file 1: Table S3).

### Bland–Altman

**Fig. 2:**
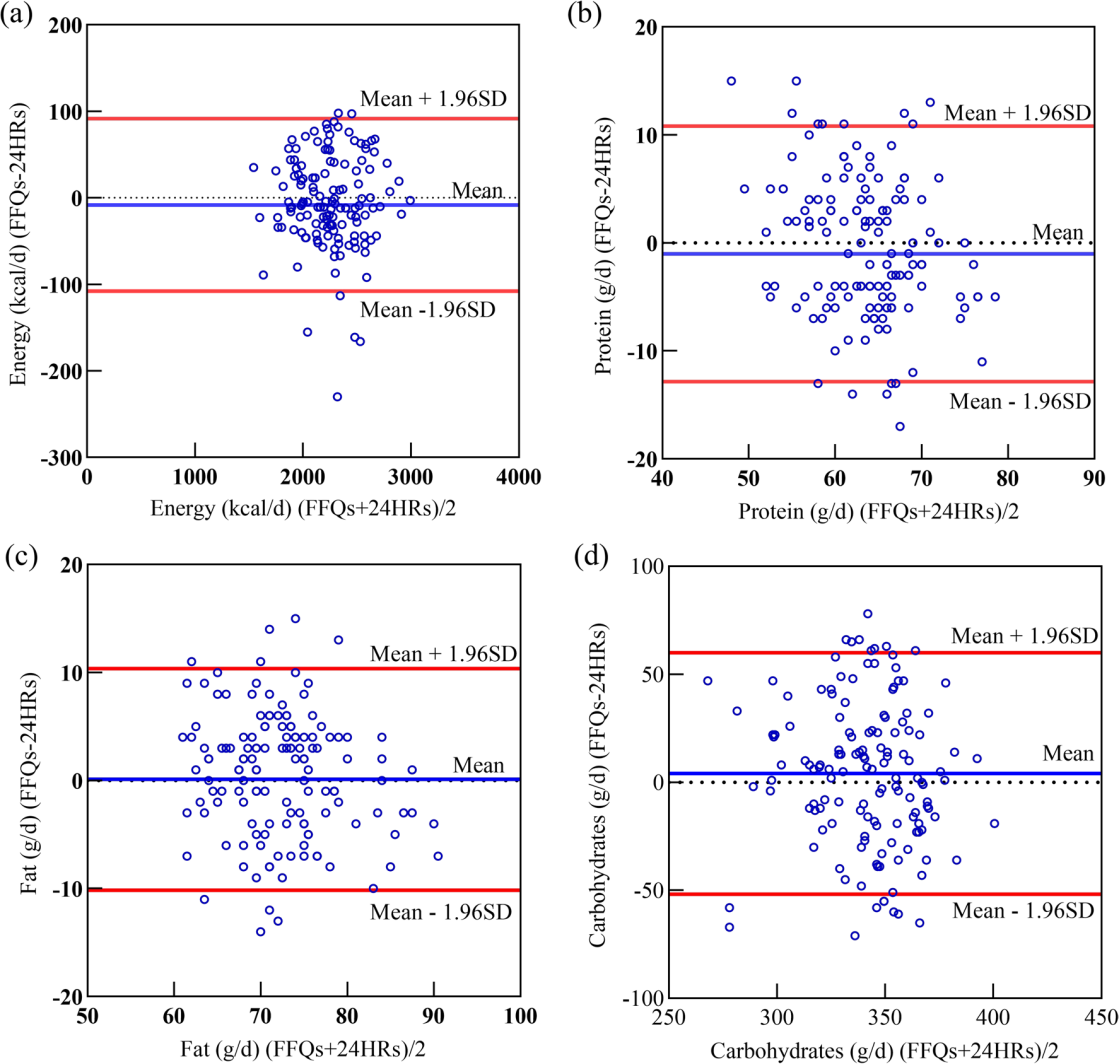
Bland–Altman plots showing agreement between the average of food frequency questionnaires (FFQs) and the three-day 24-h recalls (24HRs) in estimating the intake of energy and nutrients: **a** Energy in Kcal, **b** protein in grams, **c** fat in grams, and **d** carbohydrates in grams. (SD—standard deviation)

The results of Bland–Altman plots are shown in Fig. 2. For energy, protein, fat, and carbohydrate, the relationship was directly visualized between differences and means. There was no linear trend between the differences and means. Approximately all the points dropped on a range of the 95% limits of agreements (LOAs).

## Discussion

There have been many developed FFQs for certain populations so far, but few studies have focused on multi-ethnic groups. A 109-item FFQ was adapted to assess the daily intake of foods and nutrients among multi-ethnic backgrounds in northwest China. The results showed reasonable reproducibility and moderate validity for all nutrients.

According to the correlation analysis of nutrients between SCC, ICCs, and weighted kappa roughly above 0.5 [28], the FFQ presented acceptable reliability to estimate the daily dietary consumption of participants. These observed values were similar to or slightly higher than other studies, in which correlation coefficients generally ranged from 0.27 to 0.69 for nutrients [6, 10, 13]. The time interval of measurements could influence the reliability. The distinct interval of 3 to 24 months was conducted when testing the reproducibility of FFQ, 3 months [6], 9 months [13], and one year [10]. Longer interval of an interview might underestimate reliability due to the risk of diet habit variation. Conversely, a shorter interval between two FFQs tended to accept repeat responses, thus overestimating relevance. In this study, FFQ1 and FFQ2 were conducted three months apart, which may give contributed to reliability but proved to be a suitable interval [21].

To some extent, FFQ provided higher estimates of partial nutrients compared to 24HRs. Similar to the study of Australia [29], their FFQ slightly over-assessed 66% nutrients intake compared to weighed food records. One possible explanation was that 24HR could ignore the consumption of certain seasonal food, those daily intakes may vary considerably due to a high changing frequency. The relative validity evaluated via FFQ against 24HR was similar to reliability. The Pearson correlation coefficients assessed strong agreement between energy and nutrients, about 78.6% and 74.1% of the unadjusted and adjusted values were above 0.4, respectively. Generally speaking, the higher the values, the higher the correlation for nutrients. Former studies that suggested an acceptable correlation threshold relate to correlation coefficients above 0.4–0.5 [21, 30]. The validity results of our FFQ coincided with other major cohorts [31, 32]. Moreover, the validate coefficients were similar to studies assessing validity among multi-ethnic settings in foreign countries; 0.48–0.61 [33], 0.24–0.46 [34], and 0.57–0.74 [35].

In comparison with validity investigations in China, a study conducted among the rural Chinese populations showed values from 0.39 to 0.68 [9]. Zang et al. demonstrated a high validity with 0.33–0.77 in Shanghai [11]. The length of food list is a crucial factor in determining the questionnaires' accuracy and relative viability. Some FFQs were as short as 37 ~ 50 items [5, 7], which could avoid subject fatigue while limiting their options maximally. As a result, estimations of intakes will be inaccurate, and energy intake will either be over- or under-reported. Traditional FFQs include more than one hundred sorts of items. The length and format of FFQ depend on the purpose of the study, food availability and variability among the specific populations. Our FFQ consisting of 109 food items is considered to be reasonable [21], as an area with low food variability due to special geographical environments and Muslim culture. Furthermore, the form of interview-administered measurement contributed to the validity improvement.

Among all nutrients, the higher energy-adjusted correlation coefficients were cholesterol (0.75) and fats (0.68). A possible factor was that native residents prefer high-calorie foods such as roast meat, beef, and mutton [36] due to the more developed animal husbandry, which led to an overestimated validity that may be attributed to their frequent consumption and easily remembered by individuals. Similar results on the validation of all macronutrients and most micronutrients were significantly good or acceptable (vitamin A, thiamin, phosphorus, iodine, sodium, and riboflavin), but some micronutrients were poor, especially iron and copper [37]. According to the statistics of large cohort studies [38, 39], the dietary iron intake of Chinese residents is generally 19.7–23 mg/d. It seems that the iron intake in the study was lower than the national average. Since the Chinese diet is largely plant-based, the intake of non-heme iron can be used as a proxy for the intake of grains, vegetables, and fruits [38]. Combined with the level of food groups in this study, most of the subjects' dietary iron was non-heme iron, and the low intake of vegetables and fruits in the Xinjiang population could be partially explained. The copper levels in our current study (median: 0.7–1.0 mg/day) were close to the dietary reference intake for copper (0.9 mg/day for adults). The seasonal availability of food may be a factor in the weaker correlations of FFQs across shorter time periods.

Among different ethnic groups, the *Han* group (median: 0.53) tended to have a lower validity than the ethnic minority group (median: 0.57). Different from the explanation of education differences caused lower validation [40], *Han* groups tended to have more proportion of the higher education level across total groups. However, those who were well educated may be affected by greater social desirability bias [41]. This might due to specific differences in the diet and lifestyles of the multi-ethnic populations in Xinjiang. Food preferences for certain foods were associated with religious customs, and pork intake was not reported by Muslims. For religious culturally sensitive, those minorities would be able to skip the section on specific meat items. The findings of the current FFQ validation indicated that there is room for improvement in the estimate of iron and copper.

Due to the variety of local cuisines in China, especially in multi-ethnic settings of Xinjiang province, it is harder to estimate the portion size of dietary intake information precisely using FFQ than in other populations with single diet background [42]. Cade et al. [21] found that the agreement between FFQs and a reference dietary method was higher when portion size specified on the questionnaire (0.4–0.5) compared with no portion size specified (use of average portion weights to compute intakes: 0.2–0.5). Other scholars [43] hold the same opinion and emphasized common portion sizes for estimating nutrient intake more accurately. Open-ended questions rather than describing an accurate portion intend to decline the validity [44]. Consistent with our findings, food items with certain sizes on the semi-quantitative food frequency questionnaire always show more accurate results [8]. According to Freedman [45], the recalling bias, individual variation in the process of administration, and inaccurate portion size estimation are possible reasons for the low magnitude of correlation. Although 24HR may not fully reflect the overall dietary intakes over a long time, approximately 75% of validation studies conducted it as a reference method due to the accuracy and easier administration to capture daily intakes across a varied diet [21]. In addition, 24HR was sufficiently independent from FFQs due to distinct sources of errors, which rely on a long-time or short-time memory, respectively. Scholars generally believe that using a weighing method might obtain more relatively reliable results on the size and weight of food. Nevertheless, the methods undoubtedly were time-consuming and labor-intensive, and it is difficult to promote on a large scale. These methods above both have their own advantages and disadvantages, and which one to choose should combine with practical applications.

This study has strengths and limitations that need to be described. To our knowledge, it was the first comprehensive validation study of dietary assessment among randomly assembled multi-ethnic adult populations in northwest China, setting the criteria for adults under 65 years of age, and the actual age distribution of the included population ranged from 20 to 65 years. Therefore, we believe that there is a certain representation of adults in the Xinjiang region. Our FFQ was adapted to include a comprehensive list of both traditional and novel foods, providing an accepted evaluation of relative dietary intakes. In summary, the results on the agreement and effectiveness of our FFQ were generally satisfactory and not inferior to other investigations among more homogeneous groups. Whereas, further validation should be conducted on bigger scales of populations derived from diverse multi-ethnic backgrounds.

The study still has some limitations. Indeed, selection bias cannot be avoided in hospital-based studies. However, the study objects are random hospital physical examination population, most of which are similar to the normal population structure in the community, so the selection error is small, and the results may be representative of a wide range of people. Dietary sodium intake is low compared to other studies. Over- and underestimations in sodium intake have been similarly reported by various studies. On the one hand, there may be several errors related to the FFQ such as errors in individuals’ reports, different sodium content in food items, and daily alterations in diet [25]. Besides, precise measurement of sodium intake is rather challenging, due to the diverse distribution of sodium in foods [46]. In the following study, we will further enhance the questionnaire's items and assess its consistency with serum indicators such as 24-h urine sodium.

Willett [47] has recommended reasonable sample size of 100 to 200 for validation studies. Our sample size is in line with the requirements, there still is a need to recruit larger samples of individual ethnic subgroups to support the validation results. However, measuring short-term dietary intake by three days was also considered an acceptable design to capture the usual intake [48]. The investigation should be carried out including weekdays and weekends in each season to minimize individual variation and seasonal diet differences to capture the dietary habits throughout a long period even the whole year [49]. Additionally, without a comparison of quality assessment tool, like biomarkers. It is well known that dietary recall only reflects short-term dietary intake. Biomarkers are probably an alternative reference method for estimating nutrients intake, including 24 h urinary sodium and nitrogen [50]. However, it is difficult to eliminate the complex affection of the absorption and metabolism of nutrients in the body [51]. Biomarkers may become indicators of short-term consumption and formulating strategies to obtain measures of long-term intake remains a challenge [52]. Furthermore, the accuracy of biomarkers needs various prospective and retrospective study validation.

## Conclusions

In this study, we developed an FFQ that provides a novel, comprehensive, and culture-sensitive method for dietary assessment. The result of acceptable reproducibility and moderate validity indicates that this FFQ is an appropriate tool for nutrition epidemiological exploration among the multi-ethnic populations in northwest China.

### Supplementary Information

**Additional file 1. Table S1.** List of main food items and food groups of FFQ. **Table S2.** Daily nutrient intakes and FFQ reproducibility and validity among Han group. **Table S3.** Daily nutrient intakes and FFQ reproducibility and validity among ethnic minority group.

## Data Availability

The datasets supporting the conclusions of this article are included within the article and its additional file.

## Abbreviations

FFQ: Food frequency questionnaire
24HR: 24-Hour recall
BMI: Body mass index
LOAs: Limits of agreement
ICC: Intra-class correlation coefficients
SD: Standard deviation
