# Supplementary material for: Reliability and validity of a semi-quantitative food frequency questionnaire: dietary intake assessment among multi-ethnic populations in Northwest China
Source: J Health Popul Nutr. 2023 Oct 19;42:111. doi: 10.1186/s41043-023-00452-9 (PMC10585915; doi:10.1186/s41043-023-00452-9)
Supplement: Supplementary file 1 — Additional file 1. Table S1. List of main food items and food groups of FFQ. Table S2. Daily nutrient intakes and FFQ reproducibility and validity among Han group. Table S3. Daily nutrient intakes and FFQ reproducibility and validity among ethnic minority group. [file 41043_2023_452_MOESM1_ESM.docx]

| **Supplemental Table S1**. List of main food items and food groups of FFQ | | |
| --- | --- | --- |
| Main food items and food groups | Items number | Food items |
| Cereals | 14 | rice; rice pilaf; porridge; rice noodles; naan; steamed bread; Chinese noodles/ fried noodles; bread; meat nan; steamed stuffed bun (steamed bag/thin leather bag); dumplings/wontons; noodles soup; pancakes; instant noodles |
| Coarse grains | 6 | corn; corn paste; tubers (potato/sweet potato/yam/taro); millet; oats; fried food (fried dough sticks/flax balls/fritters); |
| Fresh vegetables | 17 | orange-yellow vegetables (tomatoes/peppers/ pumpkin/carrot); light color vegetables (cucumber/beansprouts/eggplant); dark vegetables (leeks/oilseed vegetables/canola/spinach); other dark green vegetables (broccoli, beans); carrot/green radish/white radish; green onions, garlic; pickled vegetables (pickles/sauerkraut) |
| Mushroom | 4 | the mushroom; black fungus; needle mushroom; sea algae (kelp/laver) |
| Fresh fruit | 10 | apple; pear; orange; banana; watermelon; peach; grapes; cantaloupe; apricot; pomegranate |
| Poultry and meat | 14 | fat/lean beef; fat/lean lamb; fat/lean pork; barbecue; blood clot; animal viscera(heart/tongue/stomach/intestine); duck; chicken; goose; pigeon; shrimp; fish; other aquatic products (squid/sea cucumber); processed meat (sausage/bacon/preserved meat) |
| Eggs and Dairy products | 4 | milk; yogurt; egg; other eggs (duck egg/goose egg/quail egg) |
| Legumes and soy products | 4 | chickpea; dried beans (mung beans/yellow beans/red beans); soy milk; tofu (bean skin/soy chicken) |
| Nuts | 8 | peanut; melon seeds; walnut; almonds; raisins; red jujube; rose sauce; honey |
| Water and drinks | 10 | beer; red wine; liquor; tea; plain water; carbonated drinks; fruit juice drinks; milk tea; coffee; sports drinks |
| Snacks/desserts | 9 | chocolate; puffed food; wafer; hot pot balls (meatballs/fish ball); candy; cake; biscuit; ice cream; butter |
| Condiments | 9 | flour; rice; vegetable oil; animal oil; plant oil mix; salt; sugar; vinegar; barbeque sauce |
| Total | 109 |  |

| **Supplemental Table S2.** Daily nutrient intakes and FFQ reproducibility and validity among *Han* group (n=62) | | | | | | | | | | | | | | | | |
| --- | --- | --- | --- | --- | --- | --- | --- | --- | --- | --- | --- | --- | --- | --- | --- | --- |
| Nutrients | | FFQ | | | | 24HR | | | | SCC | | r^§^ | | | | |
|  |  | Median | | (P25, P75) | | Median | | (P25, P75) | |  | | un-adjusted^†^ | | Energy adjusted^‡^ | | |
| Energy (Kcal) | | 2209.5 | | 2069.1, 2456.8 | | 2183.3 | | 2009.6, 2354.2 | | 0.86** | | 0.81** | |  | | |
| Protein (g) | | 65.1 | | 59.7, 72.4 | | 56.8 | | 51.3, 62.6 | | 0.76** | | 0.55** | | 0.52** | | |
| Fat (g) | | 72.8 | | 67.8, 79.2 | | 69.7 | | 58.6, 80.3 | | 0.65** | | 0.71** | | 0.67** | | |
| Carbohydrates (g) | | 322.4 | | 311.5, 341.8 | | 318.6 | | 298.6, 337.6 | | 0.71** | | 0.38* | | 0.41* | | |
| Dietary fibre (g) | | 15.3 | | 12.8, 16.7 | | 14.7 | | 10.3, 17.6 | | 0.47* | | 0.24 | | 0.26 | | |
| Cholesterol (mg) | | 274.9 | | 256.3, 291.3 | | 277.5 | | 243.4, 289.6 | | 0.66** | | 0.61** | | 0.56** | | |
| Vitamin A (μg RE) | | 551.2 | | 502.1, 594.8 | | 523.5 | | 487.6, 578.9 | | 0.57** | | 0.52** | | 0.48 | | |
| Vitamin D (μg) | | 11.2 | | 8.7, 14.3 | | 10.3 | | 8.1, 13.2 | | 0.46* | | 0.75** | | 0.78** | | |
| Vitamin E (mg) | | 13.6 | | 12.1, 14.8 | | 14.5 | | 10.9. 18,7 | | 0.62** | | 0.26 | | 0.21 | | |
| Vitamin K (μg) | | 68.3 | | 53.6, 74.7 | | 68.7 | | 56.4, 77.9 | | 0.46* | | 0.63** | | 0.61** | | |
| Thiamin (mg) | | 0.9 | | 0.6, 1.2 | | 0.9 | | 0.5, 1.4 | | 0.36* | | 0.65** | | 0.66** | | |
| Riboflavin (mg) | | 1.1 | | 0.6, 1.6 | | 0.8 | | 0.6, 1.4 | | 0.77** | | 0.56** | | 0.58** | | |
| Vitamin B6 (mg) | | 0.9 | | 0.8, 1.2 | | 1.2 | | 0.9, 1.6 | | 0.74** | | 0.68** | | 0.61** | | |
| Vitamin B12 (μg) | | 1.6 | | 0.9, 2.1 | | 1.5 | | 1.2, 1.7 | | 0.55** | | 0.75** | | 0.71** | | |
| Vitamin C (mg) | | 62.1 | | 55.2, 70.8 | | 73.2 | | 67.8, 83.4 | | 0.73** | | 0.33* | | 0.32* | | |
| Folate (μg) | | 332.7 | | 301.8, 346.6 | | 374.3 | | 324.8, 467.2 | | 0.65** | | 0.37* | | 0.34* | | |
| Calcium (mg) | | 603.6 | | 507.6, 674.2 | | 633.4 | | 405.7, 825.1 | | 0.54** | | 0.56** | | 0.58** | | |
| Phosphorus (mg) | | 732.5 | | 678.2, 765.5 | | 732.1 | | 589.5, 846.9 | | 0.82** | | 0.47** | | 0.43* | | |
| Potassium (mg) | | 1753.7 | | 1642.3, 2003.7 | | 1763.8 | | 1538.5, 1897.7 | | 0.69** | | 0.66** | | 0.63** | | |
| Sodium (mg) | | 1638.9 | | 1347.3, 1896.6 | | 1656.9 | | 1543.7, 1803.6 | | 0.68** | | 0.54** | | 0.55** | | |
| Magnesium (mg) | | 288.4 | | 263.5, 303.4 | | 297.8 | | 285.1, 318.5 | | 0.51** | | 0.23 | | 0.21 | | |
| Iron (mg) | | 13.4 | | 12.1, 15.8 | | 12.1 | | 10.3, 13.6 | | 0.61** | | 0.14 | | 0.15 | | |
| Iodine (μg) | | 87.2 | | 66.4, 106.3 | | 82.3 | | 71.4, 99.8 | | 0.42* | | 0.37* | | 0.32* | | |
| Zinc (mg) | | 13.4 | | 11.5, 16.3 | | 11.5 | | 9.8, 12.4 | | 0.65** | | 0.25 | | 0.23 | | |
| Selenium (μg) | | 64.8 | | 56.4, 72.8 | | 57.1 | | 50.4, 62.4 | | 0.66** | | 0.58** | | 0.55** | | |
| Copper (mg) | | 0.9 | | 0.6, 1.3 | | 0.8 | | 0.5, 1.5 | | 0.35* | | 0.12 | | 0.11 | | |
| Niacinamide (mg) | | 276.4 | | 261.2, 288.3 | | 276.5 | | 227.4, 289.5 | | 0.38* | | 0.57** | | 0.53** | | |
| Choline (mg) | | 452.3 | | 356.2, 503.8 | | 428.5 | | 313.5, 457.9 | | 0.47* | | 0.63** | | 0.61** | | |
| NOTE: Daily intakes of nutrients estimated by two FFQs and 24HRs: Median (P25, P75); SCC: Spearman correlation coefficients for reproducibility | | | | | | | | | | | | | | | | |
| ^§^r: Pearson correlation coefficients for relative validity | | | | | | | | | | | | | | | | |
| ^†^Based on log transformed values, ^‡^Energy adjusted using the residual method | | | | | | | | | | | | | | | | |
| Statistical significance was accepted with *P <0.05 (2-tailed). **P <0.01. | | | | | | | | | | | | | | | | |
| Reproducibility: FFQ1 *vs.* FFQ2; Validity: FFQs *vs.* 24HRs | | | | | | | | | | | | | | | | |
|  | | | | | | | | | | | | | | | | |
| **Supplemental Table S3.** Daily nutrient intakes and FFQ reproducibility and validity among ethnic minority group (n=77) | | | | | | | | | | | | | | | | |
| Nutrients | FFQ | | | | 24HR | | | | SCC | | r^§^ | | | |  |  |
|  | Median | | (P25, P75) | | Median | | (P25, P75) | |  | | un-adjusted**^†^** | | Energy adjusted^‡^ | |  |  |
| Energy (Kcal) | 2366.7 | | 2023.2, 2476.1 | | 2209.6 | | 1987.3, 2385.6 | | 0.63** | | 0.77** | |  | |  |  |
| Protein (g) | 63.4 | | 58.8, 73.4 | | 61.5 | | 53.4, 67.8 | | 0.67** | | 0.46* | | 0.45* | |  |  |
| Fat (g) | 73.8 | | 65.9, 80.4 | | 75.3 | | 60.2, 89.4 | | 0.74** | | 0.68** | | 0.66** | |  |  |
| Carbohydrates (g) | 315.7 | | 289.5, 361.7 | | 324.5 | | 278.3, 339.8 | | 0.68** | | 0.67** | | 0.68** | |  |  |
| Dietary fibre (g) | 13.8 | | 11.3, 16.2 | | 14.9 | | 10.6, 18.7 | | 0.56** | | 0.33* | | 0.28* | |  |  |
| Cholesterol (mg) | 263.5 | | 248.6, 287.5 | | 258.2 | | 231.4, 287.3 | | 0.62** | | 0.79** | | 0.67** | |  |  |
| Vitamin A (μg RE) | 534.7 | | 511.3, 573.6 | | 556.7 | | 472.2, 598.8 | | 0.79** | | 0.66** | | 0.57** | |  |  |
| Vitamin D (μg) | 12.1 | | 7.6, 15.8 | | 11.2 | | 8.3, 14.1 | | 0.59** | | 0.63** | | 0.72** | |  |  |
| Vitamin E (mg) | 13.3 | | 11.4, 15.5 | | 12.6 | | 11.2, 13.7 | | 0.45* | | 0.41* | | 0.36* | |  |  |
| Vitamin K (μg) | 65.3 | | 51.7, 73.8 | | 67.8 | | 5.4, 77.9 | | 0.76** | | 0.58** | | 0.57** | |  |  |
| Thiamin (mg) | 0.8 | | 0.7, 1.4 | | 0.9 | | 0.7, 1.5 | | 0.58** | | 0.76** | | 0.73** | |  |  |
| Riboflavin (mg) | 1.0 | | 0.7, 1.5 | | 0.9 | | 0.6, 1.3 | | 0.61** | | 0.79** | | 0.76** | |  |  |
| Vitamin B6 (mg) | 1.0 | | 0.8, 1.3 | | 1.1 | | 0.8, 1.4 | | 0.54** | | 0.76** | | 0.79** | |  |  |
| Vitamin B12 (μg) | 1.4 | | 1.1, 1.6 | | 1.6 | | 1.3, 1.8 | | 0.77** | | 0.43* | | 0.41* | |  |  |
| Vitamin C (mg) | 61.8 | | 51.2, 71.5 | | 78.9 | | 65.7, 84.6 | | 0.64** | | 0.67** | | 0.62** | |  |  |
| Folate (μg) | 365.9 | | 305.7, 405.7 | | 391.3 | | 336.2, 447.6 | | 0.48** | | 0.51** | | 0.49* | |  |  |
| Calcium (mg) | 587.6 | | 522.2, 606.5 | | 612.5 | | 553.4, 687.8 | | 0.71** | | 0.65** | | 0.63** | |  |  |
| Phosphorus (mg) | 734.6 | | 678.4, 761.4 | | 713.2 | | 582.7, 821.5 | | 0.68** | | 0.63** | | 0.61** | |  |  |
| Potassium (mg) | 1603.3 | | 1578.3, 1693.6 | | 1823.6 | | 1675.3, 1986.2 | | 0.35* | | 0.57** | | 0.53** | |  |  |
| Sodium (mg) | 1568.3 | | 1317.4, 1765.2 | | 1633.5 | | 1502.8, 1824.9 | | 0.48* | | 0.62** | | 0.61** | |  |  |
| Magnesium (mg) | 267.4 | | 223.4, 303.5 | | 312.6 | | 278.3, 356.9 | | 0.33* | | 0.17 | | 0.18 | |  |  |
| Iron (mg) | 12.8 | | 11.6, 15.2 | | 12.7 | | 10.7, 14.2 | | 0.68** | | 0.11 | | 0.13 | |  |  |
| Iodine (μg) | 85.9 | | 67.8, 112.3 | | 88.5 | | 77.6, 92.4 | | 0.66** | | 0.52** | | 0.49* | |  |  |
| Zinc (mg) | 11.9 | | 10.5, 13.7 | | 10.8 | | 9.3, 12.1 | | 0.49* | | 0.21 | | 0.23 | |  |  |
| Selenium (μg) | 57.4 | | 52.2, 68.9 | | 59.6 | | 51.4, 66.8 | | 0.52** | | 0.67** | | 0.63** | |  |  |
| Copper (mg) | 0.8 | | 0.7, 0.9 | | 0.9 | | 0.6, 1.5 | | 0.58** | | 0.13 | | 0.12 | |  |  |
| Niacinamide (mg) | 262.7 | | 223.4, 295.8 | | 288.8 | | 215.7, 342.8 | | 0.51** | | 0.53** | | 0.52** | |  |  |
| Choline (mg) | 461.3 | | 406.3, 528.5 | | 445.2 | | 323.8, 458.4 | | 0.68** | | 0.52** | | 0.54** | |  |  |
| NOTE: Daily intakes of nutrients estimated by two FFQs and 24HRs: Median (P25, P75); SCC: Spearman correlation coefficients for reproducibility | | | | | | | | | | | | | | | |  |
| r^§^: Pearson correlation coefficients relative validity | | | | | | | | | | | | | | | |  |
| ^†^Based on log transformed values, ^‡^Energy adjusted using the residual method | | | | | | | | | | | | | | | |  |
| Statistical significance was accepted with *P <0.05 (2-tailed). **P <0.01. | | | | | | | | | | | | | | | |  |
| Reproducibility: FFQ1 *vs.* FFQ2; Validity: FFQs *vs.* 24HRs | | | | | | | | | | | | | | | |  |
